# Comparison between staged laparoscopic technique in children with high intra-abdominal undescended testis: a systematic review and meta-analysis

**DOI:** 10.12688/f1000research.141110.2

**Published:** 2024-04-16

**Authors:** Safendra Siregar, Jupiter Sibarani, Zola Wijayanti, Albert Ciam

**Affiliations:** 1 Urology, Padjadjaran University, Bandung, West Java, Indonesia

**Keywords:** Staged laparoscopic technique, intra-abdominal undescended testes, surgery, systematic review, meta-analysis.

## Abstract

**Background:**

This study performed a systematic review and meta-analysis to compare the outcomes of the staged laparoscopic Fowler-Stephens Laparoscopic Orchiopexy (FSLO) and Staged Laparoscopic Traction Orchiopexy (SLTO) in patients with intra-abdominal testes (IAT).

**Methods:**

This study reviewed literature published from 2016 to 2024. A systematic literature search was conducted on three databases: PubMed, ScienceDirect, and Google Scholar, using keywords (High intra abdominal testis) AND (("Fowler Stephens laparoscopic orchiopexy" OR (FSLO)) OR (Staged Laparoscopic traction orchiopexy OR (SLTO)). Non-randomized trials and observational studies comparing staged laparoscopic FSLO and SLTO, without any time range restriction are included. Studies without FLSO orchidopexy as a control, case reports, case studies, duplicate publication, no full text and non-English studies are excluded. This study used the PRISMA protocol, the Jadad Scale, and the Newcastle Ottawa Scale (NOS) to evaluate the included studies. To analyze statistical data, the Review Manager (RevMan) software was used. The Chi-squared test was used to calculate statistical heterogeneity in the meta-analysis.

**Results:**

There were 240 patients from 5 studies (109 in the SLTO group and 131 FSLO in group). The primary outcome of this study is testicular descent and atrophy. There were no significant differences in testicular descent (RR:1.08[0.96 – 1.23],p<0.20,I ^2^:0%) and atrophy rate (RR:0.45[0.19 – 1.09],p<0.08,I ^2^:0%). Secondary outcomes are the duration of operation in both the first and second stages. Statistical analysis reveals a significantly lower first-stage operation time in the FSLO group (MD:9.31[7.08,11.55], p<0.05;I ^2^:94%). At the same time, lower second-stage operation times are significantly reported in the SLTO group (MD:-4.05[-7.99,-0.12],p<0.05; I ^2^:60%).

**Conclusions:**

In terms of testicular descent and testicular atrophy the SLTO technique yields similar results to the FSLO technique. Both techniques have advantages and disadvantages, and we recommend SLTO as the first choice in children with a high IAT of <4 cm.

**PROSPERO registration:**

CRD42023412407

## Introduction

Cryptorchidism is one of the most frequent congenital deformities of male neonates, also known as undescended testis (UDT). The incidence varies according to gestational age, affecting 1.0–4.6% of full-term infants and 1.1–45% of preterm infants. ^1^ Undescended testis (UDT), also known as cryptorchidism, is a condition in which the testicles are not ordinarily found at the bottom of the scrotum. ^2^ For clinical reasons, UDT is usually classified between palpable and non-palpable. The management of UDT is determined by the location and presence of the testes. About 20% of UDT are non-palpable, and about 10-50% of these impalpable testes either vanished or atrophied. ^3^

UDT diagnosis is established by clinical examination, and its diagnosis is supplemented by imaging techniques, for example, B-ultrasound and magnetic resonance imaging; however, laparoscopic exploration remains the gold standard for unpalpable UDT. ^4^^–^^6^ Undescended intra-abdominal testis (IAT) is a type of non-palpable UDT that occur in 40% of laparoscopy findings. ^1^ Short gonadal arteries typically make it difficult to successfully mobilize the testis to the scrotum without strain, making IAT a technical surgical challenge for pediatric urologists. ^7^ Usually, a High IAT that lies more than 2 cm superior to the internal inguinal ring will probably not reach the scrotum and requires a staged procedure. ^1^

The most effective and safest procedure for highly impalpable IAT is laparoscopic staged orchiopexy. ^8^ Several techniques have been described for laparoscopic orchidopexy. After spermatic vascular transection, single-stage testicular descent fixing was carried out, as Fowler and Stephens first described in 1959. ^9^ The Fowler-Stephens laparoscopic orchiopexy (FSLO) procedure describes proximal cutting and transection of the testicular vessels with preservation of the collateral arterial blood supply through the deferential artery and cremasteric vessels to gain sufficient length, which helps mobilize and relocate the testis into the scrotum. ^1^^,^^5^^,^^6^^,^^9^ Due to the nature of these procedures, the testicles may be in jeopardy of hypotrophy or atrophy if the collateral blood supply is lacking. ^1^ In one-stage FSLO, the testicular survival rate ranges between 50 and 65%, while in second-stage FSLO, it is 90%. ^1^

Shehata *et al*. ^10^ proposed a preliminary study with the preservation of a vascular technique called SLTO (staged laparoscopic traction orchiopexy) to overcome these problems. This technique does not divide the testicular vessels, which spares the major testicular blood supply. The strategy is based on tissue expansion of the testicular vasculature to expand the distended veins to the opposing anterosuperior part of the iliac spine without tearing them apart. ^10^

However, it is still debated whether SLTO techniques have more advantages than FSLO. ^11^ Currently, few studies compare head-to-head measures between FSLO and SLTO with small sample sizes. The best approach and schedule for the treatment of undescended testes have been a matter of debate for decades, ^12^ and recent studies showed sub-optimal results in managing high IAT. In the scientific community, coming to a consensus is difficult, particularly in the medical domain, where one needs to have a long enough follow-up in a randomized controlled study with a big enough size and standardized patient groups. ^11^ In this study, we executed a systematic review and meta-analysis to compare the staged laparoscopic FSLO and SLTO outcomes in patients with intra-abdominal testes.

## Methods

### Search strategy

A systematic literature search was performed in electronic databases, including PUBMED, ScienceDirect, and Google Scholar, from 2016 to 2024. The following keywords used are as follows: (High intra abdominal testis) AND ((“Fowler Stephens laparoscopic orchiopexy” OR (FSLO)) OR (Staged Laparoscopic traction orchiopexy OR (SLTO)). References and reviews were also applied to broaden the search.

The reporting is written based on Preferred Reporting Items for Systematic Reviews and Meta-Analyses (PRISMA) guidelines. ^28^ The International Prospective Register of Systematic Review (PROSPERO) study’s Registration Number CRD42023412407 on 8 ^th^ April 2023,

### Study selection

In our systematic review and meta-analysis, three reviewers screened title and abstracts independently. Non-randomized trials and observational studies comparing staged laparoscopic FSLO and SLTO, without any time range restriction are included. Studies without FLSO orchidopexy as a control, case reports, case studies, duplicate publication, no full text and non-English studies are excluded.

### Data extraction

Three reviewers independently screened each eligible study and each report retrieved to decide whether a study met the inclusion criteria of the review and in the case of a deadlocked vote, a fourth reviewer was asked to make the final decision. The variables obtained from the articles including the first author’s name, year of publication, type of study, sample size, the definition of IAT, mean age, testicular descent, testicular atrophy, mean operation time, time of follow-up, slippage, number of operators, additional finding and period waiting time from stage 1 to stage 2 from each indicvidual study will be displayed in table. Study intervention characteristics and comparing against the planned groups will be tabulated for each synthesis. The main outcome measures of our analysis were to assess the successful descent defined by testicular descent and testicular atrophy. Secondary outcome measures included the duration of the operation. Any missing data will be discarded, and only available data will be analyzed.

### Assessment of study quality

We evaluated the quality of the meta-analysis studies using the Jadad Composite Scale and Newcastle Ottawa Scale (NOS) ( Table 1). The results showed a good quality four-point scale in Jadad Composite Scale in a prospective randomised study and eight points in NOS for three retrospective studies.

**Table 1.** Jadad Scale and Newcastle Ottawa Scale (NOS) for Bias Tools.

Article	Study design	Quality assessment	
		Jadad Scale	Newcastle Ottawa Scale
Dawood *et al*., 2021	Single Center, Randomized Prospective Study	4	-
Liu *et al*., 2021	Single Center, Retrospective Cohort	-	8
Bawazir *et al*., 2021	Single Center, Retrospective Cohort	-	8
Alekrasshy *et al*., 2023	Single Center, Retrospective Cohort	-	8
Valeska *et al.*, 2024	Multi Center, Retropective Cohort	-	8

### Statistical analysis

Review Manager (RevMan) version 5.3 were used to analyze the data. We computed mean differences (MDs) with 95% confidence intervals for continuous data (CIs). Relative Risk (RR) was shown for dichotomous data. Algebraic manipulation to convert reported statistic to required statistic or effect estimate may be performed if needed. The Chi-squared test with significance set at P<0.05 was used to determine the statistical heterogeneity in the meta-analysis, and the I ^2^ statistic was used to determine the heterogeneity. With significant heterogeneity I ^2^, a random-effect model was used. Otherwise, the fixed-effect model was used.

Subgroup and sensitivity analyses will be conducted to explore possible causes of heterogeneity among study results assess robustness of the synthesized results when needed.

## Results

### Literature search

**Figure 1.**
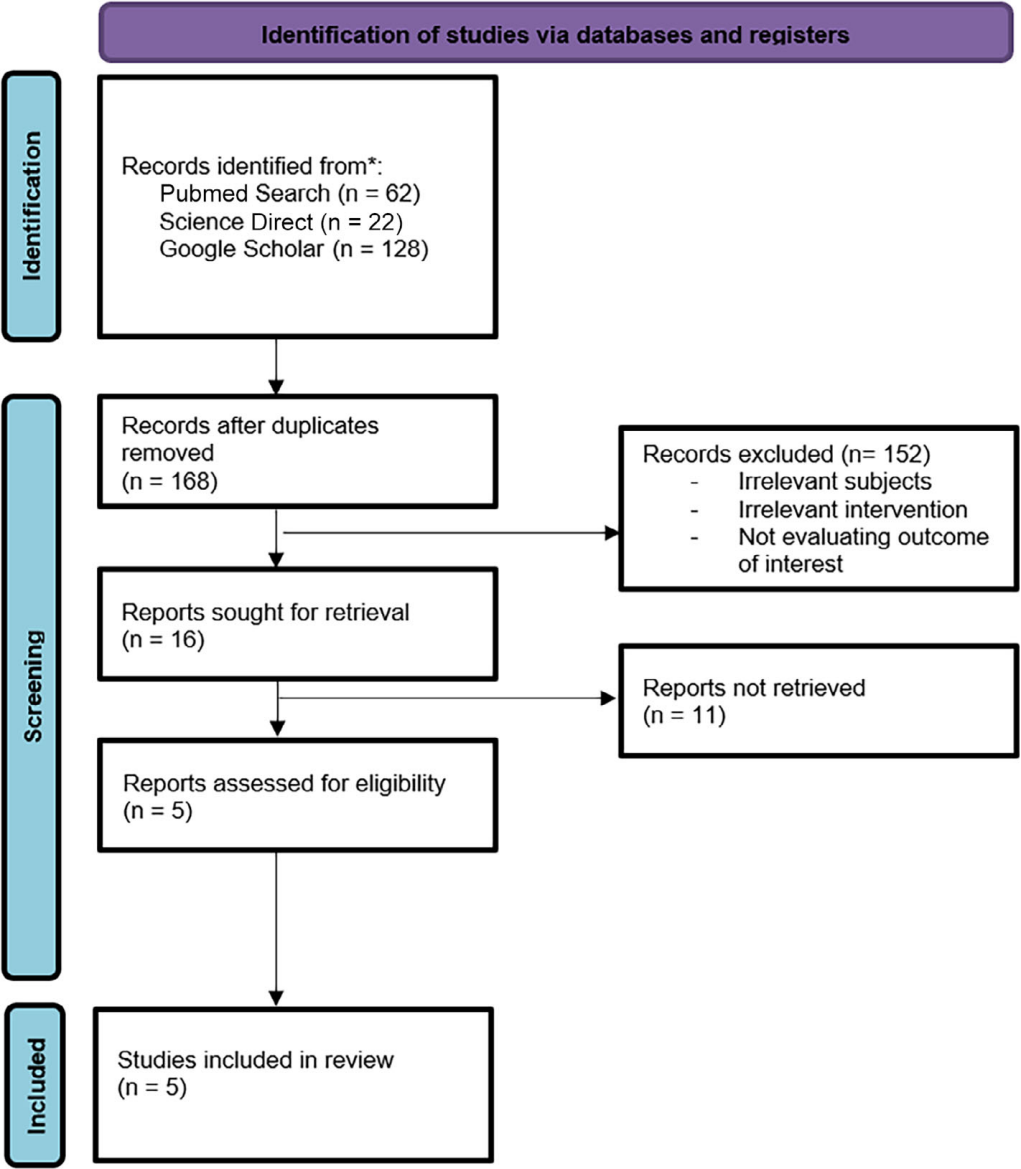
Literature search strategy according to PRISMA guideline.

After article screening and applying exclusion criteria, 212 articles were identified from the three databases. A total of 147 studies were examined after duplicates were eliminated. Sixteen of these papers with reviewed full texts were determined to be relevant based on their titles and abstracts. A total of five papers were finally included in the qualitative and quantitative analysis ( Figure 1).

### Study characteristics

We identified 212 studies, and the PRISMA protocol was implemented. After removing duplicates and excluding studies with irrelevant subjects, irrelevant intervention, and studies not evaluating our outcome of interest, we yielded five studies from which 240 patients (109 SLTO group and 131 FSLO group) with IAT m *et al*l inclusion requirements and were included in this meta-analysis and systematic review. One RCT that was appraised using the Jadad scale had good quality. Nonetheless, the remaining four studies were cohort studies and had a fair quality score on the Newcastle-Ottawa scale ( Table 1). Study characteristics, including the first author’s name, year of publication, type of study, sample size, the definition of IAT, mean age, testicular descent, testicular atrophy, mean operation time, time of follow-up, slippage, number of operators, additional finding and period waiting time from stage 1 to stage 2 are shown in Table 2.

**Table 2.** Study characteristics.

Journal		Dawood *et al.*, 2021	Liu *et al.*, 2021	Bawazir *et al.*, 2021	Alekrasshy *et al.*, 2023	Bidault-Jourdainne *et al.,* 2024
Type of Study		Single Center, Randomized Prospective Study	Single Center, Retrospective Cohort	Single Center, Retrospective Cohort	Single Center, Retrospective Cohort	Multi Center, Retrospective Cohort
Sample Size		21 using FSLO, 20 using Shetata group	22 in FLSO group, 21 in Shetata group	18 in FSLO group, 11 in Shetata Group	18 in FSLO group, 20 in Shetata group	42 in FS group, 31 in Shetata group
Definition of IAT		the testis, >2 cm from the ipsilateral inner ring	the testis, >2 cm from the ipsilateral inner ring	the testes >1 cm or higher above the ring	NA	The testes >3 cm above the internal inguinal ring
Mean Age	SLTO	32.6 ± 22.3 (month)	15.30 ± 2.38 (month)	20.27 ± 10.57 (month)	2.7 ± 0.4 (years)	15.6 ± 46.4 (months)
	FSLO	29.5 ± 4.6 (month)	15.38 ± 2.14 (month)	24.39 ± 17.53 (month)	2.5 ± 1.9 (years)	13.1 ± 38.2 (months)
Testicular Descent	SLTO	13/20 (65%)	22/22 ( 100%)	8/11 ( 72.73%)	18/20 (90%)	31/37 (83.8%)
	FSLO	14/21 ( 66.6%)	19/23 (82.60%)	11/18 (61.11%)	15/18 (83.3%)	42/52 (80.8%)
Testis Atrophy	SLTO	0/20 (0%)	0/22 (0%)	0/11 (0%)	0/20 (0%)	4/37 (11.8%)
	FSLO	3/21 (14.2%)	1/23 (4.3%)	3/18 (16.6%)	2/18 (11.1%)	6/52 (11.5%)
Operation Time (Mins)	SLTO	Stage 1 44.2 ± 5.9 stage 2 41.9 ± 7.3	Stage 1 62.75 ± 6.02 Stage 2 60.41 ± 5.36	stage 1 58. ± 9.39 Stage 2 76.33±12.23	Stage 1 62.19 ± 33.8 Stage 2 81.42 ± 11.3	NA
	FSLO	Stage 1 31.7 ± 4.6 stage 2 35.4 ± 6	Stage 1 63.57 ± 5.78 Stage 2 60.90 ± 5.13	stage 1 34.61 ± 6.43 Stage 2 74.18±12.62	Stage 1 35.71 ± 14.6 Stage 2 74 ± 06.21	NA
Follow up		6 months	6-48 months	12 months	12 months	3 and 12 months
Number of Operator		2	1	NA	NA	NA
Additional Finding	Post operative hematoma	NA	NA	1 patient	NA	NA
Period waiting time from staged 1 to staged 2	SLTO	6 months	6 months	6 months	6 months	2 months
	FSLO	6 months	3 months	3 months	3 months	6 months

Notes: SLTO, Staged Laparoscopic Traction Orchiopexy; FSLO, Fowler-Stephens Laparoscopic Orchiopexy; IAT, Undescended intra abdominal testis.

### Primary outcome

**Testicular descend**

**Figure 2.**
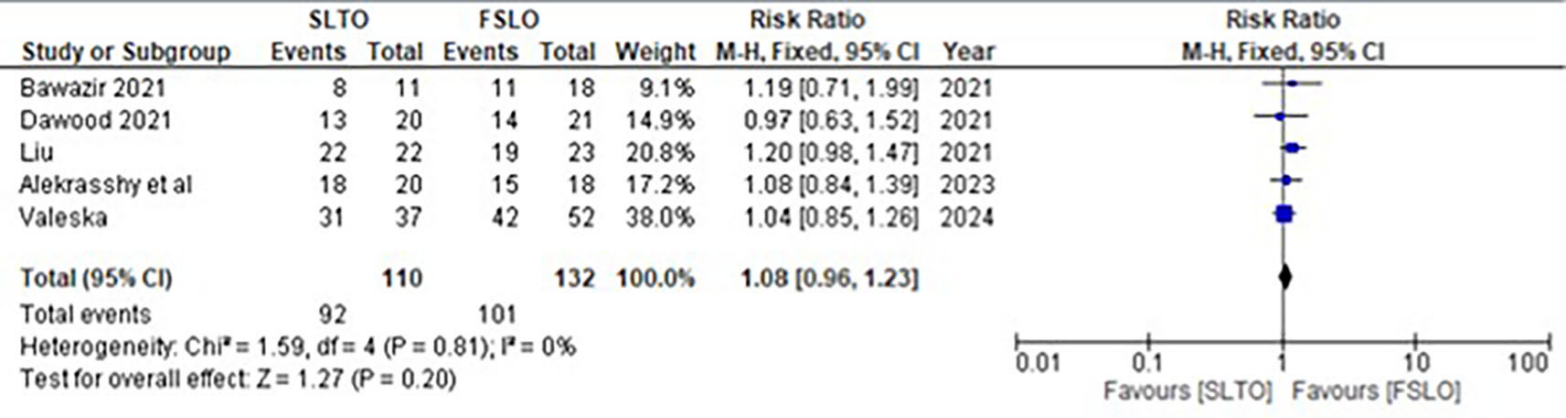
Forrest plot of testicular descend.

The testicular ascend success rate for both FSLO and STLO was reported in all five studies. The testicular growth rate between the STLO and FSLO groups had low heterogeneity and was not statistically significant, according to a meta-analysis of these trials (RR: 1.08; 95% CI: 0.96 – 1.23; p<0.20) ( Figure 2).

**Testicular atrophy**

**Figure 3.**
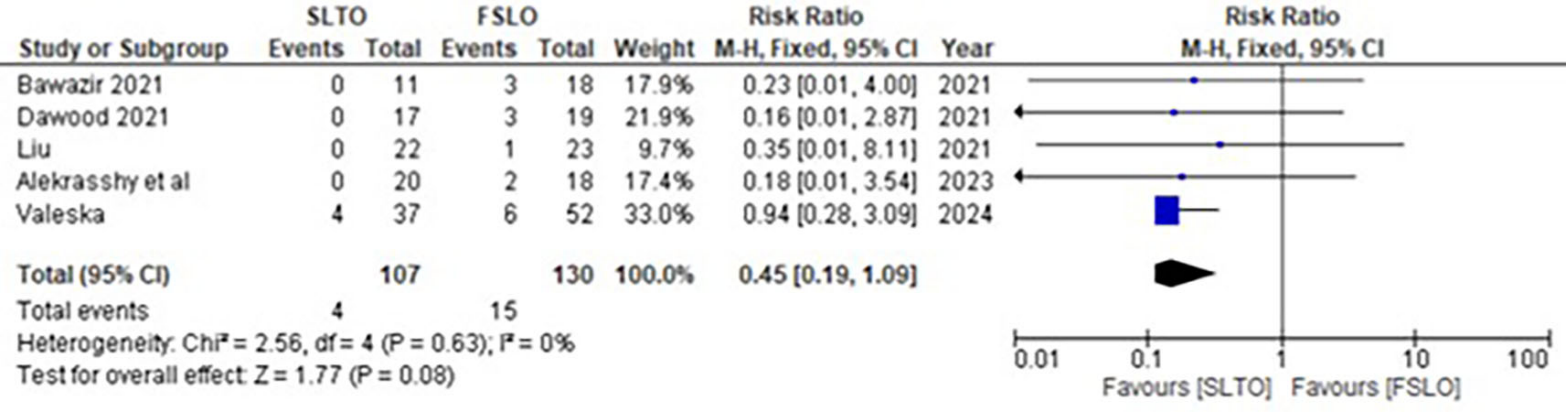
Forest plot of testicular atrophy.

The testicular atrophy rate for both FSLO and STLO was recorded in all five studies. The testicular atrophy rate between STLO and FSLO groups had low heterogenicity and was not statiscally significant, according to a meta-analysis of these trials (RR: 0.45; 95% CI: 0.19 – 1.09; p<0.08, I ^2^: 0%) ( Figure 3).

### Secondary outcome

**Operation time in the first stage**

**Figure 4.**
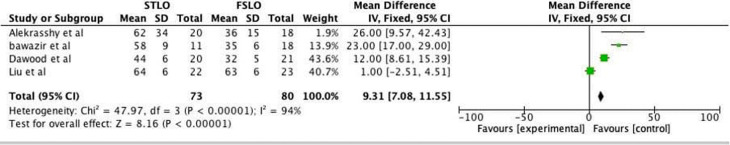
Forest plot of operation time in first stage.

The FSLO group had significantly shorter operation times (Mean Difference: 9.31; 95% CI: 7.08 – 11.55; p<0.0001), and there was considerable heterogeneity, according to a meta-analysis of these studies ( Figure 4).

**Operation time in the second stage**

**Figure 5.**
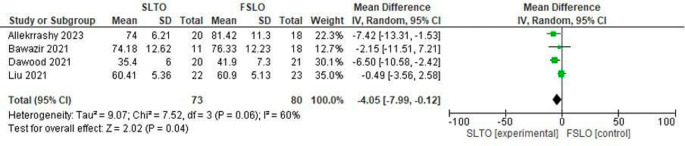
Forest plot of operation time in the second stage.

All four studies reported the mean second-stage operation time of both FSLO and STLO. The SLTO group had a statistically significant decreased operation time (Mean Difference: -4.05; 95% CI: -7.99 – 0.12; p<0.04), and there was considerable heterogeneity, according to a meta-analysis of these investigations ( Figure 5).

**Evaluation for publication bias**

Due to the small number of research, a funnel plot was not generated. Tests for funnel plot asymmetry should generally only be conducted when there are at least ten study groups. The power of the test is insufficient to distinguish between chance and actual asymmetry because of the small number of included studies examining the results of the staged laparoscopic FSLO and SLTO in patients with intra abdominal testes. ^13^

## Discussion

Pediatric surgeons continue to face many difficulties when doing surgery for the abdominal testis. Although numerous surgical techniques have been documented to address this issue, none have emerged as the clinical paradigm’s gold standard. The lack of an optimal surgical method or imaging technologies and an inadequate understanding of the interactions between the genetic, physiological, and hormonal pathways involved in testicular descent are the main causes of the unavailability of an international agreement on a management plan. ^14^^–^^19^ The most typical method of exploring unpalpable testicles is by minimally invasive laparoscopy. An orchiopexy, or the surgical transfer of the testis into the scrotum, can be carried out if the testicle is visible. The absent or vanishing testis is diagnosed if the spermatic arteries have blind endings. In this way, both the diagnosis and treatment can be performed in a single setting by laparoscopic. ^14^

There is still no consensus regarding the criteria for high IAT; however, the latest Ains shams classification type 4 ( Figure 2) can be used to categorize high IAT for similar criteria with several studies that define high intra-abdominal testis if the testis is more than 2 cm from ipsilateral side of the inner ring. ^19^^–^^21^ These studies also have taught us that the IAT less than 2 cm away from the inner ring can be lowered and attached securely to the scrotum. Other studies like Bawazir and Bagga *et al*. defined high intra-abdominal testis if the testis is >1 cm from the ipsilateral inner ring. ^15^^,^^16^ Whereas Esposito *et al*. ^18^ and Valeska *et al.* ^17^ claimed that the standard distance is 3 cm. However, Agrawal *et al*. believed that the appropriate distance is 2.5 cm. ^19^ Multi-center studies with large samples and long-term follow-up are needed for selection criteria that could be properly used as a consensus. ^20^

Since the advancement of minimally invasive surgery, there have been several methods for laparoscopic procedures in managing IAT. In this study, we compared two surgical methods (SLTO and FSLO) for the management of High IAT. As an alternative, testes can be repositioned in the scrotum and the distal testicular vessels (TVs) and can be anastomosed with the inferior epigastric vessels through the division of the proximal TVs. This procedure is known as microvascular testicular auto-transplantation. ^23^^,^^25^^,^^26^ Unfortunately, there are several issues in the supporting data for this technique. ^23^^–^^26^ It is labor-intensive (median time: 4.5 hours), time-consuming, and has a variable success rate (84–97%). ^25^

After spermatic vascular transection, single-stage testicular descent fixing was carried out, as Fowler and Stephens first described in 1959. Since then, though with only unsatisfactory outcomes, Fowler-Stephens staged orchidopexy has dominated surgical procedures. ^9^ A single or two stages procedure to lower the testis into the scrotum is necessary and is dependent on collateral blood flow from the deferential and external spermatic arteries. To provide more direct access to the scrotum, the testis can also be passed medially to the inferior epigastric arteries via the Prentiss maneuver, which involves opening the transversalis fascia. However, this approach has been linked to testicular atrophy. A phased surgery has considerably decreased the rate of testicular atrophy, which is quite common in the initial stages of the F-S technique. ^27^ By using the test for testicular ischemia, prevention has been made to ensure testicular collateral circulation. ^28^ Testicular ischemia test was done by blocking the blood vessels of the spermatic cord with some silk thread and holding them for 10 minutes. When making a knot, it should be assured that the knot can be loosened when necessary, and then see if there are visible changes in the blood supply of testicles before and after the test. It is considered appropriate for F-S surgery if there is no visible ischemic change in the testis following spermatic vascular blockage. It means that the collateral blood supply of the testicles is plentiful, and the gubernaculum’s blood supply is adequate. ^28^ However, since the impact of the testicular ischemia test on the phenomena of testicular atrophy has not been researched, everything depends heavily on the surgeon’s practices. ^20^

Shehata proposed a pilot research with encouraging findings from gradually controlled traction facilitated by laparoscopic surgery in 2008. ^10^ This method was revised and reposted later in 2016, confirming its efficacy and safety. ^21^ Similar to the Fowler Stephen group, this procedure used a comparable anesthetic technique, operation posture, and trocar position for the puncture. ^9^ An inch superior and medial to the contralateral side of the anterior superior iliac spine (ASIS) in the abdomen, the testis is secured with a round needle to the fixation location. After three months, a second-stage laparoscopic-assisted orchiopexy was scheduled. The testicles are lowered to the scrotum and secured by incision from the scrotum on the affected side in the second stage of both surgical procedures, which are both assisted by laparoscopy. ^24^ Because the internal spermatic artery, vas deferens artery, and veins were intact throughout the procedure, the Shehata approach efficiently secured the blood supply of the testis. The spermatic cord is pushed backward and downward by the gut, and the testis is stabilized in the rightful location during the first stage. The primary advantage of this approach is the ability to progressively enlarge the spermatic cord blood vessels without causing testicular ischemia, which is achieved by either chronic intestinal compression or continual respiratory movement. ^24^ This may be explained by the SLTO technique of non-transection of the testicular vasculature and the vascular pedicle’s progressive, gentle elongation.

The successful outcome of the orchiopexy is the study’s main objective. The right testicular location in the scrotum without atrophy or ascent was considered successful. A testis in the bottom or middle of the scrotal cavity without tension was referred to as being in the scrotal position. A testis located at the neck of the scrotum was not considered a successful example since it did not meet the criteria of the scrotal location. A testicle relocation outside the scrotal cavity is referred to as testicular ascension. Based on physical examination by clinicians and the data from ultrasonography measurements, postoperative testicular atrophy was defined as the presence of a nubbin or more than 50% testicular volume loss or a postoperative testicular volume of 25% of the volume of the contralateral testis, with no detectable blood flow as evidenced by color Doppler ultrasound in comparison to the normal contralateral side.

According to our research, both groups’ testicular descent rates are equivalent. Regarding the fixed position of the scrotum in the case of High IAT, both staged laparoscopic methods showed a similar success rate. ^27^ However, because the testicular artery was disrupted during this treatment, the FSLO group has a greater rate of testicular atrophy. We believe that keeping the testicular arteries intact may result in superior outcomes and reduce the chance of testicular atrophy. When excessive tension was not applied from the beginning in the case of testicular veins that are too short, the testicular ascent would occur and could not accomplish adequate elongation necessary for free testis mobilization into the scrotum. ^22^ However, according to Aljubaibi *et al.*, the success rate of the SLTO group was 93% for <2 cm; 78% for 2-4 cm; nil for >4 cm, respectively. Hence, they advocated the FSLO procedure be conducted to testes more than 4 cm from the IIR. ^23^

The secondary outcomes of our study are mean operation time between the first and second stages of both procedures. Although it is shown that the first stage favoured the FSLO procedure, the limited number of the study showed high heterogenicity. Further comparisons are needed to conclude since SLTO is still a new technique, and a learning period is still needed for surgeons to adapt to this technique. Testicular ischemia test in FSLO also can be considered for extra 10 minutes of procedure, making both results similar is conducted. According to our review, FSLO and SLTO shared similar results in the second procedure. This may contribute to similar procedures in both techniques. This outcome is crucial for surgeons to choose which technique based on their knowledge and the comorbidities of the patient as both technique are not significantly superior in terms of testicular descend and testicular atrophy.

The success rate of the SLTO procedure for aged less than two years or distances less than 4 cm was higher than FSLO. The limitation of Shehata is that when it is applied on patients over two years, and the distance is over 4 cm, the failure rate of the Shehata increases as older age results in a longer pull distance. To measure the length gain indirectly, Shehata *et al.*, introduced a Meryland and allowed it to extend the testicular pedicle under its weight. They then measured the length from the pedicle to the anterior abdominal wall in the midline and found a mean gain of 4.7 cm. ^21^ But Aljunaibi *et al*. found a mean gain of 3.4 cm using a more objective and trustworthy method because it is connected to and evaluated against the scrotal distance. ^23^ However recent study by Valeska et al. ^17^ concluded that the age of the patient did not have any influence on the final testicular position in their study.

Another benefit was that SLTO only required three months to go from stage one to stage two, while FSLO took at least six months. Following Fowler Stephen’s recommendation, Shehata and his coauthors established a 12-week gap between the two stages. This gap is essential to facilitate neovascularization between the artery of the vas and the distal testicular arteries, which provides an alternative blood supply to the testis. Still, a recent study by Aljunaibi showed that traction orchiopexy should adhere to the same time intervals as other traction and stretching procedures used in other pathologies, such as tissue expanders and long-gap esophageal atresia. ^9^^,^^21^^,^^23^ Both approaches follow a 1-week break; then, a reevaluation was done to see if further stretching to a new stretch point is necessary after tissue remodeling has gone into effect and the preceding stretch point is no longer effective. ^23^

A shorter time between the two stages reduces the risk of internal hernia or suture slippage. It also lessens the probability that the testicles would adhere to the abdominal wall and helps to mobilize them if they do so because they are fibrinous adhesions rather than fibrous ones. By the time of follow-up, all patients had achieved the previously defined success. None of them experienced a hernia or a wound infection within proximity of the port. ^23^

The small sample size and the excessive heterogeneities in the mean operation time variables are the study’s drawbacks. Consequently, the writers believe that a more extensive study should be carried out in urology centers with a larger sample size and with surgeons who specialise in both FSLO and SLTO in the management of High IAT. Because the method was published in 2016, it is also impossible to know the fertility results for the SLTO group. So, at this time, it is impossible to compare the long-term outcomes of the two types of surgery.

In conclusion, the SLTO showed a similar result to the FSLO technique in terms of testicular descent and testicular atrophy. There is a difference in mean operation time in the first stage; however, further study is needed to conclude. The mean operation time in the second stage is shorter. Therefore, it could be inferred that the SLTO technique has superiority in terms of complication and waiting time between procedures. Both techniques have their respective advantages and disadvantages; however, we recommend SLTO as the first choice in children with high IAT < 4 cm.

## Data Availability

All data underlying the results are available as part of the article and no additional source data are required. Figshare: PRISMA checklist for ‘Comparison between staged laparoscopic technique in children with high intra abdominal undescended testis: a systematic review and meta-analysis. 10.6084/m9.figshare.24197760.v1. ^29^ Data are available under the terms of the Creative Commons Attribution 4.0 International license (CC-BY 4.0).
