# Supplementary material for: Comparison between staged laparoscopic technique in children with high intra-abdominal undescended testis: a systematic review and meta-analysis
Source: F1000Res. 2024 Apr 16;12:1511. Originally published 2023 Nov 27. [Version 2] doi: 10.12688/f1000research.141110.2 (PMC11080546; doi:10.12688/f1000research.141110.2)
Supplement: Peer review history

# Reviewer report: Reviewer response for version 1

**DOI:** 10.5256/f1000research.154524.r241646

**Published:** 2024-05-08

**Authors:** Jie Liu

**Affiliations:** 1 Yijishan Hospital of Wannan Medical College, Wuhu, China

This is an interesting study on high intra-abdominal undescended testis, and the authors conclude: In terms of testicular descent,  the SLTO technique yields similar results to the FSLO technique. The SLTO position is preferable to the FSLO position.  Both techniques have advantages and disadvantages,  and we recommend SLTO as the first choice in children with a high IAT of 4 cm. The conclusions of this study are basically the same as our actual observations, which is an interesting study. The discussion section is also well written and worth reading for researchers.

Are the rationale for, and objectives of, the Systematic Review clearly stated?

Yes

Is the statistical analysis and its interpretation appropriate?

Yes

Are sufficient details of the methods and analysis provided to allow replication by others?

Yes

Are the conclusions drawn adequately supported by the results presented in the review?

Yes

Reviewer Expertise:

pediatric surgery

I confirm that I have read this submission and believe that I have an appropriate level of expertise to confirm that it is of an acceptable scientific standard.

# Reviewer report: Reviewer response for version 1

**DOI:** 10.5256/f1000research.154524.r241648

**Published:** 2024-02-20

**Authors:** Valeska Bidault-Jourdainne

**Affiliations:** 1 Pediatric urovisceral and thoracic surgery, Hospices Civils de Lyon, Lyon, Auvergne-Rhône-Alpes, France

This article presents a systematic review of clinical studies comparing Fowlers Stephens laparoscopic two stage orchiopexy (FSLO) and Shehata laparoscopic traction staged orchiopexy (STLO), between 2016 and 2023. FSLO is the most commonly used technique for high intra-abdominal testis (IAT), but since 2008, Shehata introduced a new technique (STLO), without division of the spermatic vessels.

 4 articles were included and analysed in the review: one prospective and 3 retrospective comparative studies, with a total of 151 patients. The primary outcome analysed in the review was the testicular descend success rate, which was similar with both FSLO and STLO. Testicular atrophy was significantly lower in the STLO group (p=0.04), and completed the primary outcomes of the review. Operation times were in favour of FSLO for first stage of orchiopexy (p<0.0001), and in favour of STLO during second stage (p<0.04).

 This systematic review has a good rationale, since no consensus exists on the best technique for high IAT orchiopexy, and FSLO does not provide complete satisfaction with at least 10% testicular atrophy described in the literature. The objectives are also well exposed, although one may object that duration of each stage for orchiopexy should not be considered, if one technique is superior to the other in terms of testicular descend and atrophy.

 Methods for selection of articles and statistical analysis are well defined and adapted to the goal of this review.

 However, the small number of patients in each study included weakens the conclusion of this study. For example, only 11 patients with high IAT had STLO in Bawazir and Maghrabi's study. Thus, the conclusion with recommendations for IAT management is criticisable and lacks power. It would be more valuable to open on a larger study needed for high IAT (published recently in JPU 2024).

Are the rationale for, and objectives of, the Systematic Review clearly stated?

Yes

Is the statistical analysis and its interpretation appropriate?

Yes

Are sufficient details of the methods and analysis provided to allow replication by others?

Yes

Are the conclusions drawn adequately supported by the results presented in the review?

Partly

Reviewer Expertise:

Pediatric Urology, uro-genital surgery, testicular surgery

I confirm that I have read this submission and believe that I have an appropriate level of expertise to confirm that it is of an acceptable scientific standard, however I have significant reservations, as outlined above.

# Response

**Published:** 2024-03-26

**Authors:** Albert -

**Affiliations:** Padjadjaran University, Indonesia, Indonesia

We would like to express our gratitude for your time and efforts in reviewing our manuscript. We have carefully considered all the comments provided by the editor and reviewers. After several discussion and updates in our research data, here is our responses regarding dr Valeska comments in 2 separate points

1. ”However, the small number of patients in each study included weakens the conclusion of this study. For example, only 11 patients with high IAT had STLO in Bawazir and Maghrabi's study. Thus, the conclusion with recommendations for IAT management is criticisable and lacks power. It would be more valuable to open on a larger study needed for high IAT (published recently in JPU 2024).”

Thank you for your guidance, We have included one additional article for analysis, bringing the total to five studies involving 240 patients to strengthen our conclusion in this study and updated our pool data. Our meta-analysis revealed that there was minimal variation and no statistically significant difference in the rate of testicular growth between the STLO and FSLO groups (RR: 1.08; 95% CI: 0.96 – 1.23; p<0.20). Similarly, the rate of testicular atrophy between the STLO and FSLO groups showed low variation and lacked statistical significance (RR: 0.45; 95% CI: 0.19 – 1.09; p<0.08), with low heterogeneity (I2: 0%). In summary, the SLTO technique demonstrated comparable outcomes to the FSLO technique in terms of testicular descent and atrophy. This finding showed the importance for surgeons to make informed decisions based on patient characteristics and their own expertise, as neither technique appears significantly superior regarding testicular descent and atrophy.

1. “The objectives are also well exposed, although one may object that duration of each stage for orchiopexy should not be considered, if one technique is superior to the other in terms of testicular descend and atrophy.”

Since the result of our study show that there are no significant differences between both techniques in terms of testicular descent and testicular atrophy, we can conlude that there are no superiority in both techniques. we still need more data regarding the operation time in each stages of procedure to draw conclusion since our result show high heterogenicity mean operation time.

 Thank you

# Reviewer report: Reviewer response for version 1

**DOI:** 10.5256/f1000research.154524.r241655

**Published:** 2024-02-09

**Authors:** Ferdinando Agresta

**Affiliations:** 1 ULSS2 Marca Trevigiana, Vittorio Veneto, Treviso, Italy

well done and interesting paper to read and to know about. The analysis is well done, as is the research. The topic is interesting and important and the conclusion of the paper seems in according with and light of the actual evidence.  Again: well done systemic review and meta analysis

Are the rationale for, and objectives of, the Systematic Review clearly stated?

Yes

Is the statistical analysis and its interpretation appropriate?

Yes

Are sufficient details of the methods and analysis provided to allow replication by others?

Yes

Are the conclusions drawn adequately supported by the results presented in the review?

Yes

Reviewer Expertise:

Laparoscopy; emergency, abdominal wall

I confirm that I have read this submission and believe that I have an appropriate level of expertise to confirm that it is of an acceptable scientific standard.
